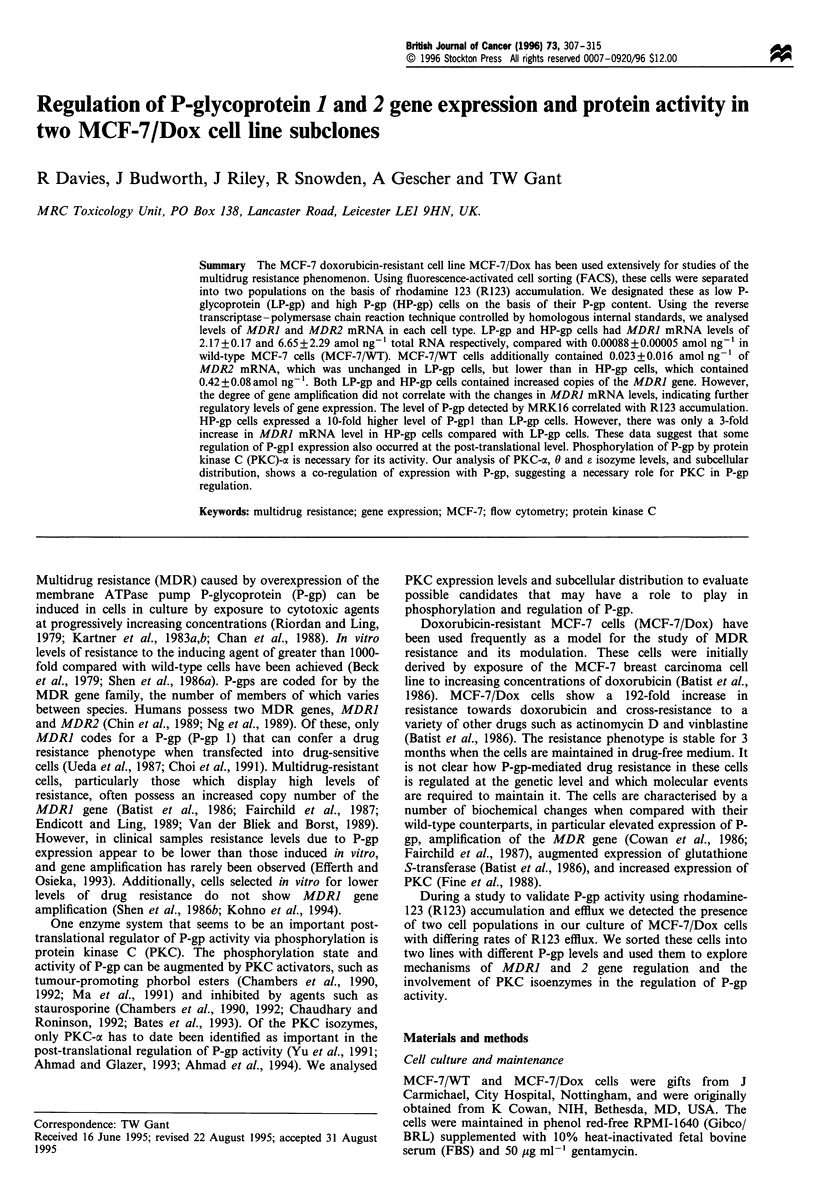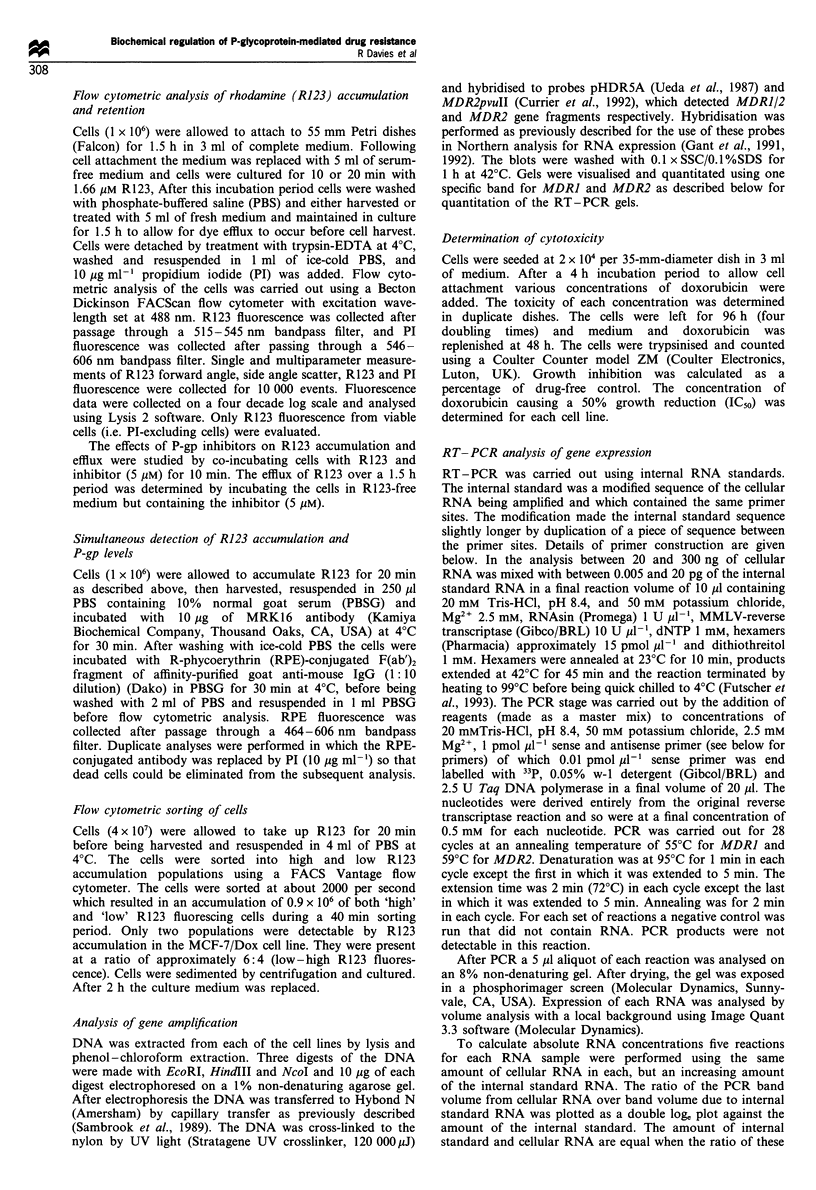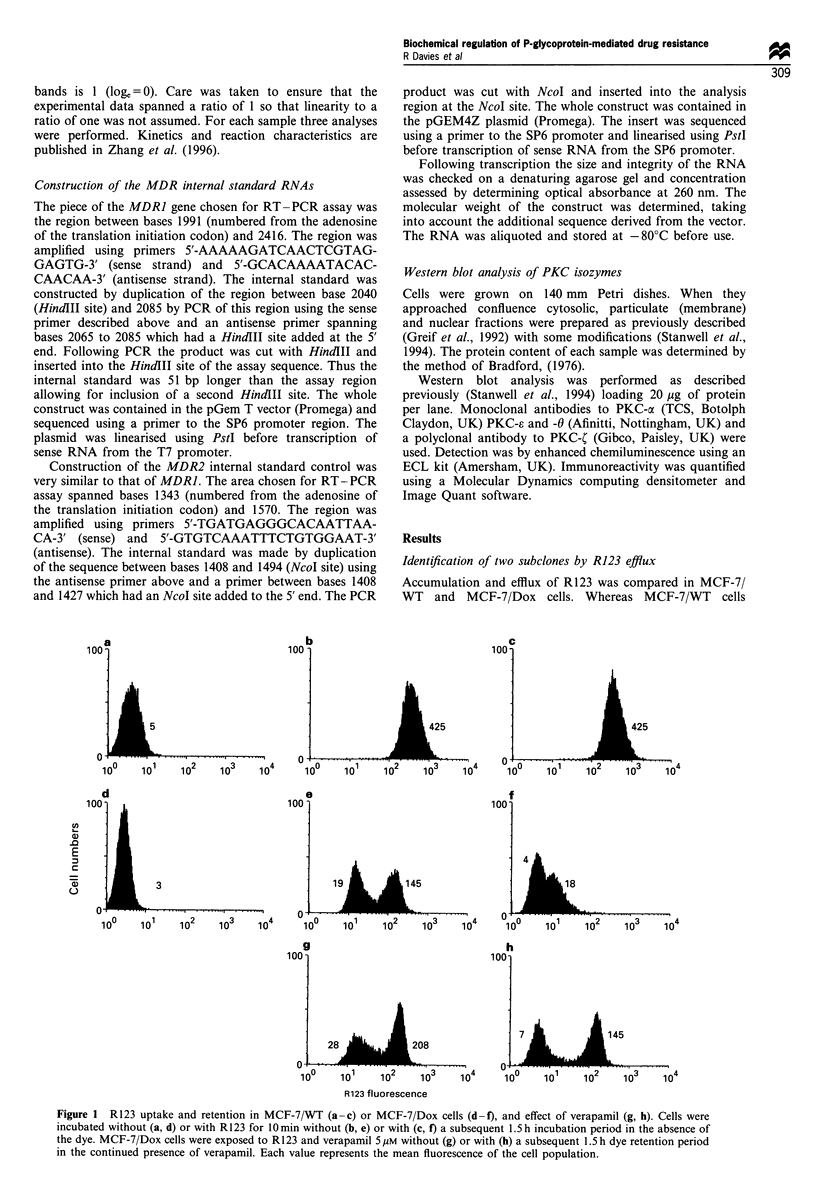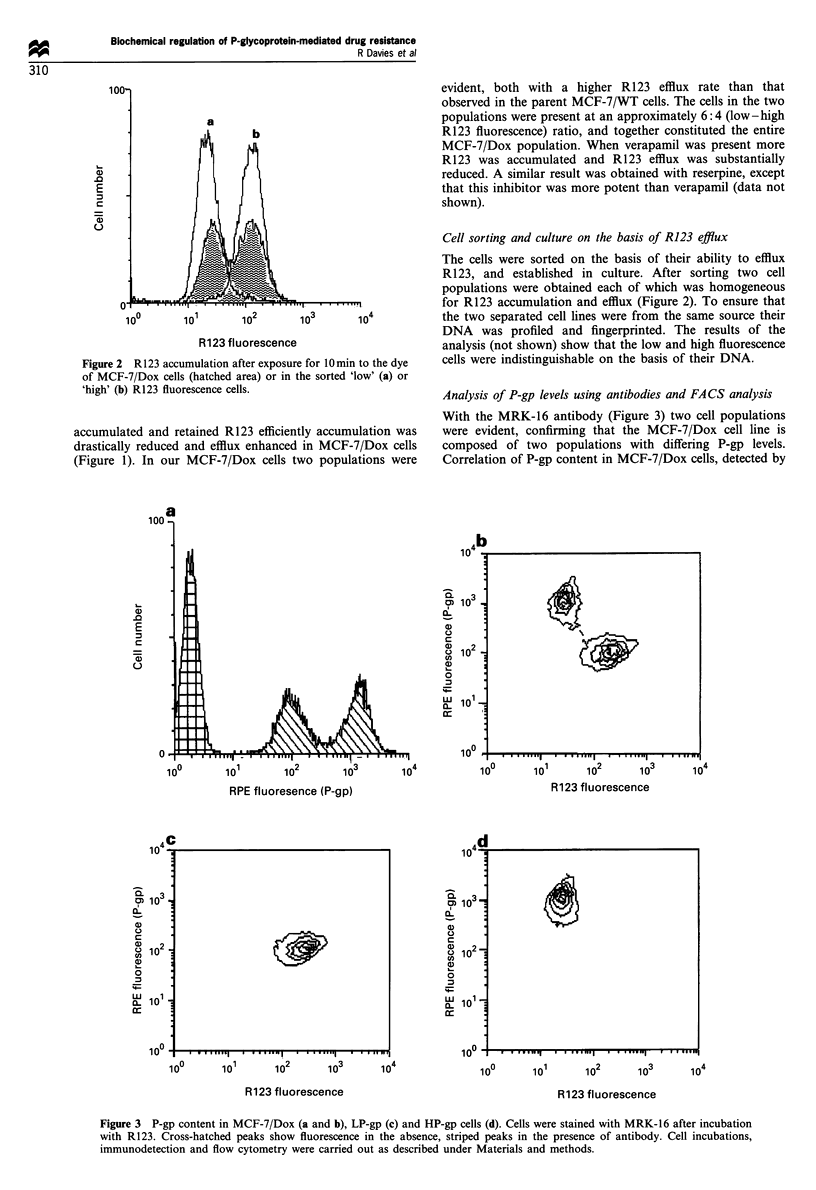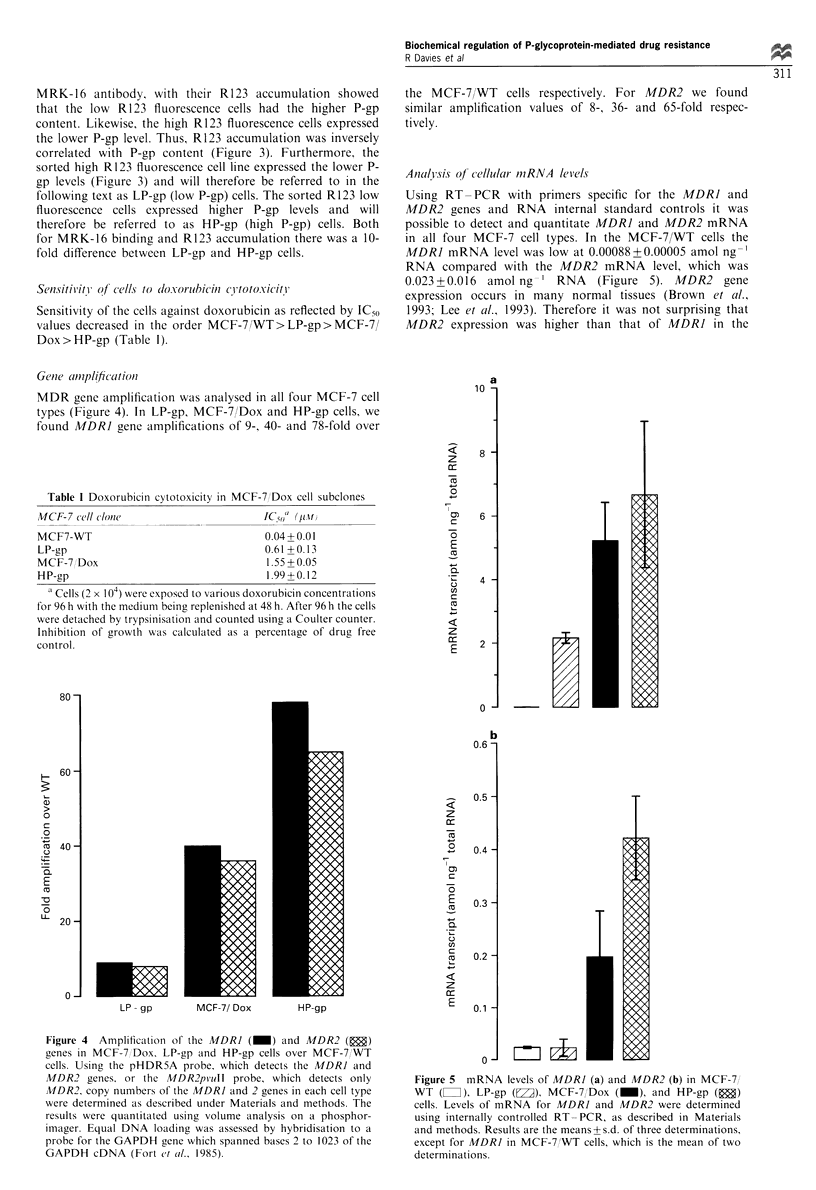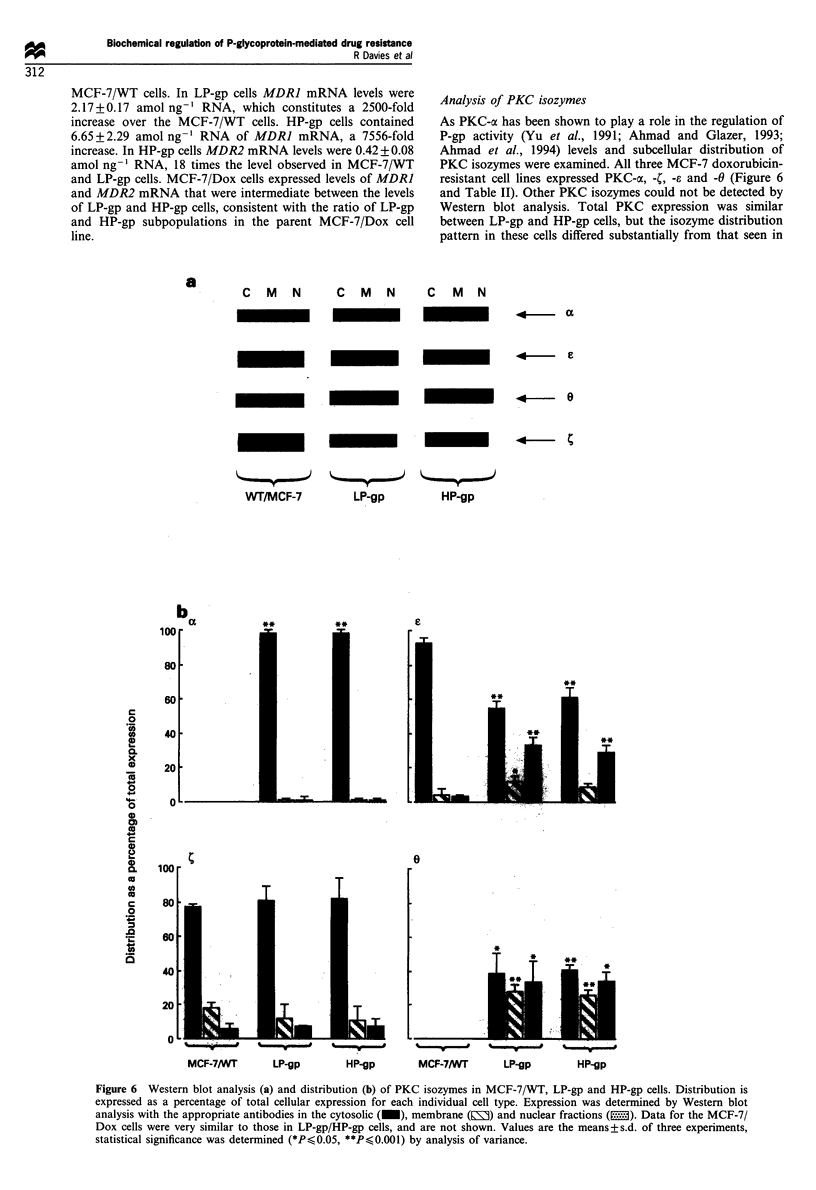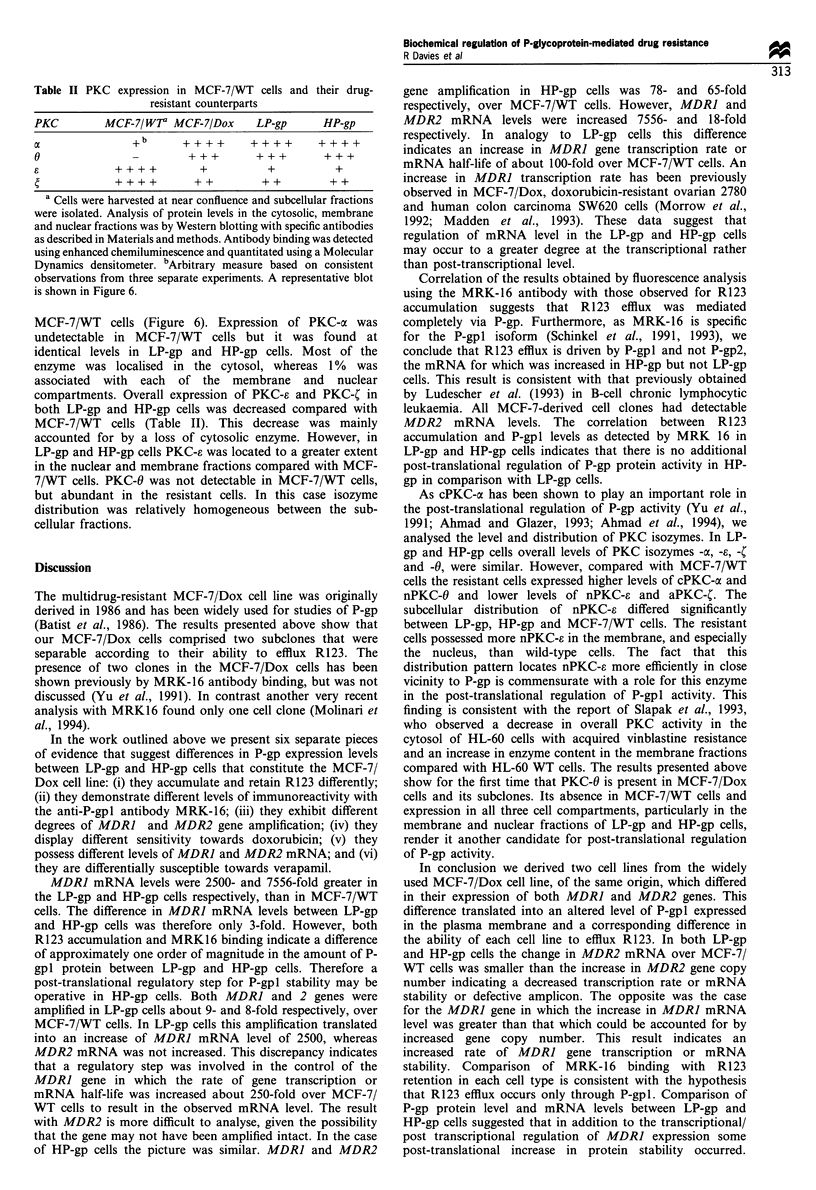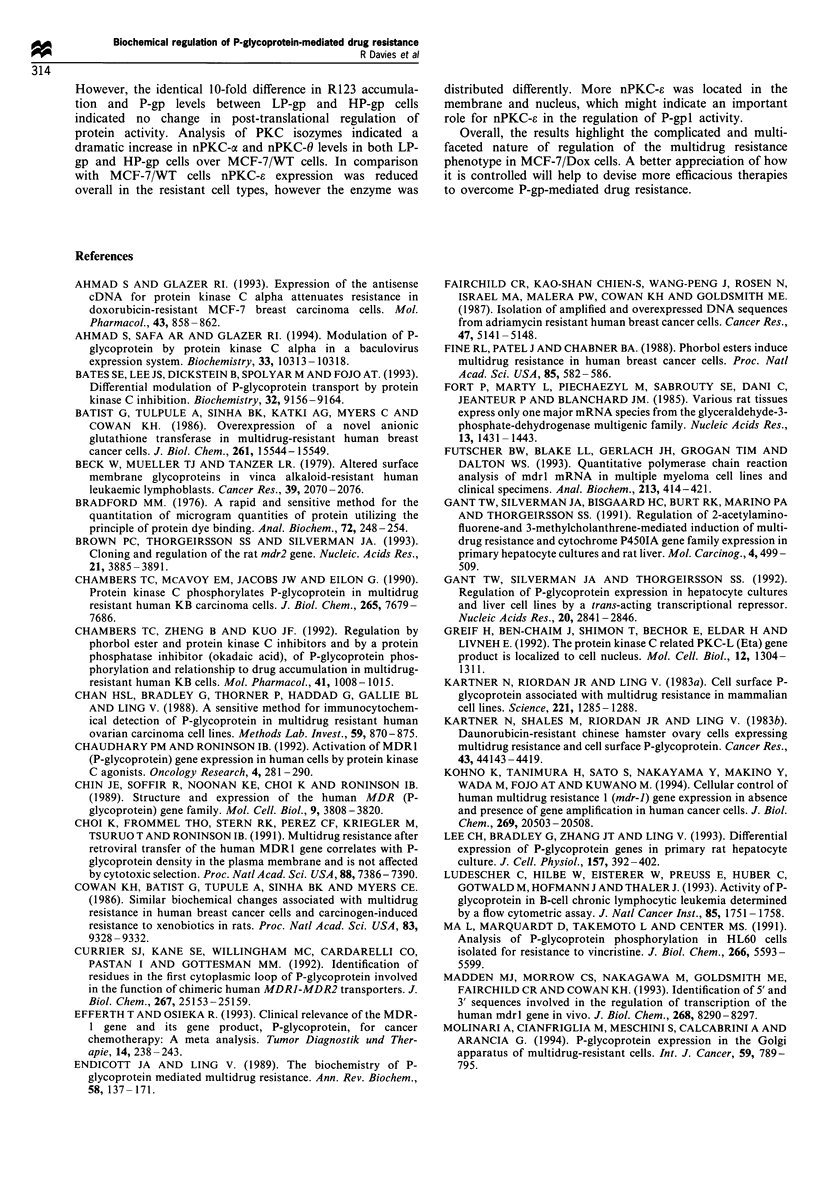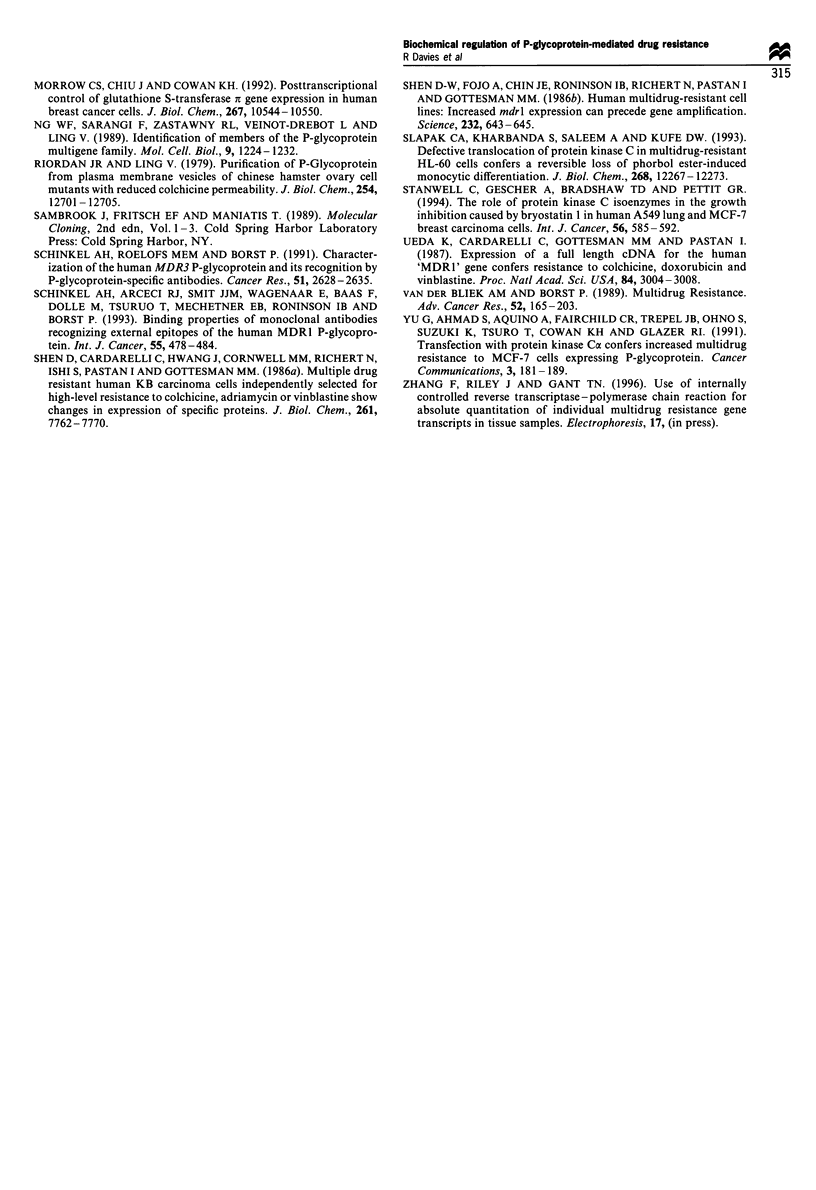# Regulation of P-glycoprotein 1 and 2 gene expression and protein activity in two MCF-7/Dox cell line subclones.

**DOI:** 10.1038/bjc.1996.54

**Published:** 1996-02

**Authors:** R. Davies, J. Budworth, J. Riley, R. Snowden, A. Gescher, T. W. Gant

**Affiliations:** MRC Toxicology Unit, Leicester, UK.

## Abstract

**Images:**


British Journal of Cancer (1996) 73, 307-315
© 1996 Stockton Press All rights reserved 0007-0920/96 $12.00

### Regulation of P-glycoprotein *1* and *2* gene expression and protein activity in two MCF-7/Dox cell line subclones

R Davies, J Budworth, J Riley, R Snowden, A Gescher and TW Gant

*MRC Toxicology Unit, PO Box 138, Lancaster Road, Leicester LE1 9HN, UK.*

**Summary** The MCF-7 doxorubicin-resistant cell line MCF-7/Dox has been used extensively for studies of the multidrug resistance phenomenon. Using fluorescence-activated cell sorting (FACS), these cells were separated into two populations on the basis of rhodamine 123 (R123) accumulation. We designated these as low P-glycoprotein (LP-gp) and high P-gp (HP-gp) cells on the basis of their P-gp content. Using the reverse transcriptase–polymersase chain reaction technique controlled by homologous internal standards, we analysed levels of *MDR1* and *MDR2* mRNA in each cell type. LP-gp and HP-gp cells had *MDR1* mRNA levels of $2.17 \pm 0.17$ and $6.65 \pm 2.29$ amol $ng^{-1}$ total RNA respectively, compared with $0.00088 \pm 0.00005$ amol $ng^{-1}$ in wild-type MCF-7 cells (MCF-7/WT). MCF-7/WT cells additionally contained $0.023 \pm 0.016$ amol $ng^{-1}$ of *MDR2* mRNA, which was unchanged in LP-gp cells, but lower than in HP-gp cells, which contained $0.42 \pm 0.08$ amol $ng^{-1}$. Both LP-gp and HP-gp cells contained increased copies of the *MDR1* gene. However, the degree of gene amplification did not correlate with the changes in *MDR1* mRNA levels, indicating further regulatory levels of gene expression. The level of P-gp detected by MRK16 correlated with R123 accumulation. HP-gp cells expressed a 10-fold higher level of P-gp1 than LP-gp cells. However, there was only a 3-fold increase in *MDR1* mRNA level in HP-gp cells compared with LP-gp cells. These data suggest that some regulation of P-gp1 expression also occurred at the post-translational level. Phosphorylation of P-gp by protein kinase C (PKC)-$\alpha$ is necessary for its activity. Our analysis of PKC-$\alpha$, $\theta$ and $\varepsilon$ isozyme levels, and subcellular distribution, shows a co-regulation of expression with P-gp, suggesting a necessary role for PKC in P-gp regulation.

**Keywords:** multidrug resistance; gene expression; MCF-7; flow cytometry; protein kinase C

Multidrug resistance (MDR) caused by overexpression of the membrane ATPase pump P-glycoprotein (P-gp) can be induced in cells in culture by exposure to cytotoxic agents at progressively increasing concentrations (Riordan and Ling, 1979; Kartner *et al.*, 1983*a,b*; Chan *et al.*, 1988). *In vitro* levels of resistance to the inducing agent of greater than 1000-fold compared with wild-type cells have been achieved (Beck *et al.*, 1979; Shen *et al.*, 1986*a*). P-gps are coded for by the MDR gene family, the number of members of which varies between species. Humans possess two MDR genes, *MDR1* and *MDR2* (Chin *et al.*, 1989; Ng *et al.*, 1989). Of these, only *MDR1* codes for a P-gp (P-gp 1) that can confer a drug resistance phenotype when transfected into drug-sensitive cells (Ueda *et al.*, 1987; Choi *et al.*, 1991). Multidrug-resistant cells, particularly those which display high levels of resistance, often possess an increased copy number of the *MDR1* gene (Batist *et al.*, 1986; Fairchild *et al.*, 1987; Endicott and Ling, 1989; Van der Bliek and Borst, 1989). However, in clinical samples resistance levels due to P-gp expression appear to be lower than those induced *in vitro*, and gene amplification has rarely been observed (Efferth and Osieka, 1993). Additionally, cells selected *in vitro* for lower levels of drug resistance do not show *MDR1* gene amplification (Shen *et al.*, 1986*b*; Kohno *et al.*, 1994).

One enzyme system that seems to be an important post-translational regulator of P-gp activity via phosphorylation is protein kinase C (PKC). The phosphorylation state and activity of P-gp can be augmented by PKC activators, such as tumour-promoting phorbol esters (Chambers *et al.*, 1990, 1992; Ma *et al.*, 1991) and inhibited by agents such as staurosporine (Chambers *et al.*, 1990, 1992; Chaudhary and Roninson, 1992; Bates *et al.*, 1993). Of the PKC isozymes, only PKC-$\alpha$ has to date been identified as important in the post-translational regulation of P-gp activity (Yu *et al.*, 1991; Ahmad and Glazer, 1993; Ahmad *et al.*, 1994). We analysed PKC expression levels and subcellular distribution to evaluate possible candidates that may have a role to play in phosphorylation and regulation of P-gp.

Doxorubicin-resistant MCF-7 cells (MCF-7/Dox) have been used frequently as a model for the study of MDR resistance and its modulation. These cells were initially derived by exposure of the MCF-7 breast carcinoma cell line to increasing concentrations of doxorubicin (Batist *et al.*, 1986). MCF-7/Dox cells show a 192-fold increase in resistance towards doxorubicin and cross-resistance to a variety of other drugs such as actinomycin D and vinblastine (Batist *et al.*, 1986). The resistance phenotype is stable for 3 months when the cells are maintained in drug-free medium. It is not clear how P-gp-mediated drug resistance in these cells is regulated at the genetic level and which molecular events are required to maintain it. The cells are characterised by a number of biochemical changes when compared with their wild-type counterparts, in particular elevated expression of P-gp, amplification of the *MDR* gene (Cowan *et al.*, 1986; Fairchild *et al.*, 1987), augmented expression of glutathione *S*-transferase (Batist *et al.*, 1986), and increased expression of PKC (Fine *et al.*, 1988).

During a study to validate P-gp activity using rhodamine-123 (R123) accumulation and efflux we detected the presence of two cell populations in our culture of MCF-7/Dox cells with differing rates of R123 efflux. We sorted these cells into two lines with different P-gp levels and used them to explore mechanisms of *MDR1* and *2* gene regulation and the involvement of PKC isoenzymes in the regulation of P-gp activity.

### Materials and methods

### *Cell culture and maintenance*

MCF-7/WT and MCF-7/Dox cells were gifts from J Carmichael, City Hospital, Nottingham, and were originally obtained from K Cowan, NIH, Bethesda, MD, USA. The cells were maintained in phenol red-free RPMI-1640 (Gibco/BRL) supplemented with 10% heat-inactivated fetal bovine serum (FBS) and 50 $\mu g\ ml^{-1}$ gentamycin.

Correspondence: TW Gant
Received 16 June 1995; revised 22 August 1995; accepted 31 August 1995

*Flow cytometric analysis of rhodamine (R123) accumulation and retention*

Cells ($1 \times 10^6$) were allowed to attach to 55 mm Petri dishes (Falcon) for 1.5 h in 3 ml of complete medium. Following cell attachment the medium was replaced with 5 ml of serum-free medium and cells were cultured for 10 or 20 min with 1.66 $\mu$M R123, After this incubation period cells were washed with phosphate-buffered saline (PBS) and either harvested or treated with 5 ml of fresh medium and maintained in culture for 1.5 h to allow for dye efflux to occur before cell harvest. Cells were detached by treatment with trypsin-EDTA at 4°C, washed and resuspended in 1 ml of ice-cold PBS, and 10 $\mu$g $ml^{-1}$ propidium iodide (PI) was added. Flow cytometric analysis of the cells was carried out using a Becton Dickinson FACScan flow cytometer with excitation wavelength set at 488 nm. R123 fluorescence was collected after passage through a 515–545 nm bandpass filter, and PI fluorescence was collected after passing through a 546–606 nm bandpass filter. Single and multiparameter measurements of R123 forward angle, side angle scatter, R123 and PI fluorescence were collected for 10 000 events. Fluorescence data were collected on a four decade log scale and analysed using Lysis 2 software. Only R123 fluorescence from viable cells (i.e. PI-excluding cells) were evaluated.

The effects of P-gp inhibitors on R123 accumulation and efflux were studied by co-incubating cells with R123 and inhibitor (5 $\mu$M) for 10 min. The efflux of R123 over a 1.5 h period was determined by incubating the cells in R123-free medium but containing the inhibitor (5 $\mu$M).

*Simultaneous detection of R123 accumulation and P-gp levels*

Cells ($1 \times 10^6$) were allowed to accumulate R123 for 20 min as described above, then harvested, resuspended in 250 $\mu$l PBS containing 10% normal goat serum (PBSG) and incubated with 10 $\mu$g of MRK16 antibody (Kamiya Biochemical Company, Thousand Oaks, CA, USA) at 4°C for 30 min. After washing with ice-cold PBS the cells were incubated with R-phycoerythrin (RPE)-conjugated $F(ab')_2$ fragment of affinity-purified goat anti-mouse IgG (1:10 dilution) (Dako) in PBSG for 30 min at 4°C, before being washed with 2 ml of PBS and resuspended in 1 ml PBSG before flow cytometric analysis. RPE fluorescence was collected after passage through a 464–606 nm bandpass filter. Duplicate analyses were performed in which the RPE-conjugated antibody was replaced by PI (10 $\mu$g $ml^{-1}$) so that dead cells could be eliminated from the subsequent analysis.

*Flow cytometric sorting of cells*

Cells ($4 \times 10^7$) were allowed to take up R123 for 20 min before being harvested and resuspended in 4 ml of PBS at 4°C. The cells were sorted into high and low R123 accumulation populations using a FACS Vantage flow cytometer. The cells were sorted at about 2000 per second which resulted in an accumulation of $0.9 \times 10^6$ of both 'high' and 'low' R123 fluorescing cells during a 40 min sorting period. Only two populations were detectable by R123 accumulation in the MCF-7/Dox cell line. They were present at a ratio of approximately 6:4 (low–high R123 fluorescence). Cells were sedimented by centrifugation and cultured. After 2 h the culture medium was replaced.

*Analysis of gene amplification*

DNA was extracted from each of the cell lines by lysis and phenol–chloroform extraction. Three digests of the DNA were made with *Eco*RI, *Hind*III and *Nco*I and 10 $\mu$g of each digest electrophoresed on a 1% non-denaturing agarose gel. After electrophoresis the DNA was transferred to Hybond N (Amersham) by capillary transfer as previously described (Sambrook *et al.*, 1989). The DNA was cross-linked to the nylon by UV light (Stratagene UV crosslinker, 120 000 $\mu$J) and hybridised to probes pHDR5A (Ueda *et al.*, 1987) and *MDR2pvu*II (Currier *et al.*, 1992), which detected *MDR1/2* and *MDR2* gene fragments respectively. Hybridisation was performed as previously described for the use of these probes in Northern analysis for RNA expression (Gant *et al.*, 1991, 1992). The blots were washed with 0.1 × SSC/0.1%SDS for 1 h at 42°C. Gels were visualised and quantitated using one specific band for *MDR1* and *MDR2* as described below for quantitation of the RT–PCR gels.

*Determination of cytotoxicity*

Cells were seeded at $2 \times 10^4$ per 35-mm-diameter dish in 3 ml of medium. After a 4 h incubation period to allow cell attachment various concentrations of doxorubicin were added. The toxicity of each concentration was determined in duplicate dishes. The cells were left for 96 h (four doubling times) and medium and doxorubicin was replenished at 48 h. The cells were trypsinised and counted using a Coulter Counter model ZM (Coulter Electronics, Luton, UK). Growth inhibition was calculated as a percentage of drug-free control. The concentration of doxorubicin causing a 50% growth reduction ($IC_{50}$) was determined for each cell line.

*RT–PCR analysis of gene expression*

RT–PCR was carried out using internal RNA standards. The internal standard was a modified sequence of the cellular RNA being amplified and which contained the same primer sites. The modification made the internal standard sequence slightly longer by duplication of a piece of sequence between the primer sites. Details of primer construction are given below. In the analysis between 20 and 300 ng of cellular RNA was mixed with between 0.005 and 20 pg of the internal standard RNA in a final reaction volume of 10 $\mu$l containing 20 mM Tris-HCl, pH 8.4, and 50 mM potassium chloride, $Mg^{2+}$ 2.5 mM, RNAsin (Promega) 1 U $\mu l^{-1}$, MMLV-reverse transcriptase (Gibco/BRL) 10 U $\mu l^{-1}$, dNTP 1 mM, hexamers (Pharmacia) approximately 15 pmol $\mu l^{-1}$ and dithiothreitol 1 mM. Hexamers were annealed at 23°C for 10 min, products extended at 42°C for 45 min and the reaction terminated by heating to 99°C before being quick chilled to 4°C (Futscher *et al.*, 1993). The PCR stage was carried out by the addition of reagents (made as a master mix) to concentrations of 20 mMTris-HCl, pH 8.4, 50 mM potassium chloride, 2.5 mM $Mg^{2+}$, 1 pmol $\mu l^{-1}$ sense and antisense primer (see below for primers) of which 0.01 pmol $\mu l^{-1}$ sense primer was end labelled with $^{33}P$, 0.05% w-1 detergent (Gibcol/BRL) and 2.5 U *Taq* DNA polymerase in a final volume of 20 $\mu$l. The nucleotides were derived entirely from the original reverse transcriptase reaction and so were at a final concentration of 0.5 mM for each nucleotide. PCR was carried out for 28 cycles at an annealing temperature of 55°C for *MDR1* and 59°C for *MDR2*. Denaturation was at 95°C for 1 min in each cycle except the first in which it was extended to 5 min. The extension time was 2 min (72°C) in each cycle except the last in which it was extended to 5 min. Annealing was for 2 min in each cycle. For each set of reactions a negative control was run that did not contain RNA. PCR products were not detectable in this reaction.

After PCR a 5 $\mu$l aliquot of each reaction was analysed on an 8% non-denaturing gel. After drying, the gel was exposed in a phosphorimager screen (Molecular Dynamics, Sunnyvale, CA, USA). Expression of each RNA was analysed by volume analysis with a local background using Image Quant 3.3 software (Molecular Dynamics).

To calculate absolute RNA concentrations five reactions for each RNA sample were performed using the same amount of cellular RNA in each, but an increasing amount of the internal standard RNA. The ratio of the PCR band volume from cellular RNA over band volume due to internal standard RNA was plotted as a double $\log_e$ plot against the amount of the internal standard. The amount of internal standard and cellular RNA are equal when the ratio of these bands is 1 ($\log_e = 0$). Care was taken to ensure that the experimental data spanned a ratio of 1 so that linearity to a ratio of one was not assumed. For each sample three analyses were performed. Kinetics and reaction characteristics are published in Zhang *et al.* (1996).

### *Construction of the MDR internal standard RNAs*

The piece of the *MDR1* gene chosen for RT–PCR assay was the region between bases 1991 (numbered from the adenosine of the translation initiation codon) and 2416. The region was amplified using primers 5′-AAAAAGATCAACTCGTAG-GAGTG-3′ (sense strand) and 5′-GCACAAAATACAC-CAACAA-3′ (antisense strand). The internal standard was constructed by duplication of the region between base 2040 (*Hin*dIII site) and 2085 by PCR of this region using the sense primer described above and an antisense primer spanning bases 2065 to 2085 which had a *Hin*dIII site added at the 5′ end. Following PCR the product was cut with *Hin*dIII and inserted into the *Hin*dIII site of the assay sequence. Thus the internal standard was 51 bp longer than the assay region allowing for inclusion of a second *Hin*dIII site. The whole construct was contained in the pGem T vector (Promega) and sequenced using a primer to the SP6 promoter region. The plasmid was linearised using *Pst*I before transcription of sense RNA from the T7 promoter.

Construction of the *MDR2* internal standard control was very similar to that of *MDR1*. The area chosen for RT–PCR assay spanned bases 1343 (numbered from the adenosine of the translation initiation codon) and 1570. The region was amplified using primers 5′-TGATGAGGGCACAATTAA-CA-3′ (sense) and 5′-GTGTCAAATTTCTGTGGAAT-3′ (antisense). The internal standard was made by duplication of the sequence between bases 1408 and 1494 (*Nco*I site) using the antisense primer above and a primer between bases 1408 and 1427 which had an *Nco*I site added to the 5′ end. The PCR product was cut with *Nco*I and inserted into the analysis region at the *Nco*I site. The whole construct was contained in the pGEM4Z plasmid (Promega). The insert was sequenced using a primer to the SP6 promoter and linearised using *Pst*I before transcription of sense RNA from the SP6 promoter.

Following transcription the size and integrity of the RNA was checked on a denaturing agarose gel and concentration assessed by determining optical absorbance at 260 nm. The molecular weight of the construct was determined, taking into account the additional sequence derived from the vector. The RNA was aliquoted and stored at −80°C before use.

### *Western blot analysis of PKC isozymes*

Cells were grown on 140 mm Petri dishes. When they approached confluence cytosolic, particulate (membrane) and nuclear fractions were prepared as previously described (Greif *et al.*, 1992) with some modifications (Stanwell *et al.*, 1994). The protein content of each sample was determined by the method of Bradford, (1976).

Western blot analysis was performed as described previously (Stanwell *et al.*, 1994) loading 20 $\mu$g of protein per lane. Monoclonal antibodies to PKC-$\alpha$ (TCS, Botolph Claydon, UK) PKC-$\varepsilon$ and -$\theta$ (Afinitti, Nottingham, UK) and a polyclonal antibody to PKC-$\zeta$ (Gibco, Paisley, UK) were used. Detection was by enhanced chemiluminescence using an ECL kit (Amersham, UK). Immunoreactivity was quantified using a Molecular Dynamics computing densitometer and Image Quant software.

### Results

### *Identification of two subclones by R123 efflux*

Accumulation and efflux of R123 was compared in MCF-7/WT and MCF-7/Dox cells. Whereas MCF-7/WT cells

**Figure 1** R123 uptake and retention in MCF-7/WT (**a**–**c**) or MCF-7/Dox cells (**d**–**f**), and effect of verapamil (**g**, **h**). Cells were incubated without (**a**, **d**) or with R123 for 10 min without (**b**, **e**) or with (**c**, **f**) a subsequent 1.5 h incubation period in the absence of the dye. MCF-7/Dox cells were exposed to R123 and verapamil 5 $\mu$M without (**g**) or with (**h**) a subsequent 1.5 h dye retention period in the continued presence of verapamil. Each value represents the mean fluorescence of the cell population.

**Figure 2** R123 accumulation after exposure for 10 min to the dye of MCF-7/Dox cells (hatched area) or in the sorted 'low' (**a**) or 'high' (**b**) R123 fluorescence cells.

accumulated and retained R123 efficiently accumulation was drastically reduced and efflux enhanced in MCF-7/Dox cells (Figure 1). In our MCF-7/Dox cells two populations were evident, both with a higher R123 efflux rate than that observed in the parent MCF-7/WT cells. The cells in the two populations were present at an approximately 6:4 (low–high R123 fluorescence) ratio, and together constituted the entire MCF-7/Dox population. When verapamil was present more R123 was accumulated and R123 efflux was substantially reduced. A similar result was obtained with reserpine, except that this inhibitor was more potent than verapamil (data not shown).

*Cell sorting and culture on the basis of R123 efflux*

The cells were sorted on the basis of their ability to efflux R123, and established in culture. After sorting two cell populations were obtained each of which was homogeneous for R123 accumulation and efflux (Figure 2). To ensure that the two separated cell lines were from the same source their DNA was profiled and fingerprinted. The results of the analysis (not shown) show that the low and high fluorescence cells were indistinguishable on the basis of their DNA.

*Analysis of P-gp levels using antibodies and FACS analysis*

With the MRK-16 antibody (Figure 3) two cell populations were evident, confirming that the MCF-7/Dox cell line is composed of two populations with differing P-gp levels. Correlation of P-gp content in MCF-7/Dox cells, detected by

**Figure 3** P-gp content in MCF-7/Dox (**a** and **b**), LP-gp (**c**) and HP-gp cells (**d**). Cells were stained with MRK-16 after incubation with R123. Cross-hatched peaks show fluorescence in the absence, striped peaks in the presence of antibody. Cell incubations, immunodetection and flow cytometry were carried out as described under Materials and methods.

MRK-16 antibody, with their R123 accumulation showed that the low R123 fluorescence cells had the higher P-gp content. Likewise, the high R123 fluorescence cells expressed the lower P-gp level. Thus, R123 accumulation was inversely correlated with P-gp content (Figure 3). Furthermore, the sorted high R123 fluorescence cell line expressed the lower P-gp levels (Figure 3) and will therefore be referred to in the following text as LP-gp (low P-gp) cells. The sorted R123 low fluorescence cells expressed higher P-gp levels and will therefore be referred to as HP-gp (high P-gp) cells. Both for MRK-16 binding and R123 accumulation there was a 10-fold difference between LP-gp and HP-gp cells.

*Sensitivity of cells to doxorubicin cytotoxicity*

Sensitivity of the cells against doxorubicin as reflected by $IC_{50}$ values decreased in the order MCF-7/WT > LP-gp > MCF-7/Dox > HP-gp (Table I).

*Gene amplification*

MDR gene amplification was analysed in all four MCF-7 cell types (Figure 4). In LP-gp, MCF-7/Dox and HP-gp cells, we found *MDR1* gene amplifications of 9-, 40- and 78-fold over the MCF-7/WT cells respectively. For *MDR2* we found similar amplification values of 8-, 36- and 65-fold respectively.

*Analysis of cellular mRNA levels*

Using RT–PCR with primers specific for the *MDR1* and *MDR2* genes and RNA internal standard controls it was possible to detect and quantitate *MDR1* and *MDR2* mRNA in all four MCF-7 cell types. In the MCF-7/WT cells the *MDR1* mRNA level was low at 0.00088 ± 0.00005 amol ng$^{-1}$ RNA compared with the *MDR2* mRNA level, which was 0.023 ± 0.016 amol ng$^{-1}$ RNA (Figure 5). *MDR2* gene expression occurs in many normal tissues (Brown *et al.*, 1993; Lee *et al.*, 1993). Therefore it was not surprising that *MDR2* expression was higher than that of *MDR1* in the

**Table I** Doxorubicin cytotoxicity in MCF-7/Dox cell subclones

| *MCF-7 cell clone* | $IC_{50}{}^{a}$ ($\mu$M) |
|---|---|
| MCF7-WT | 0.04 ± 0.01 |
| LP-gp | 0.61 ± 0.13 |
| MCF-7/Dox | 1.55 ± 0.05 |
| HP-gp | 1.99 ± 0.12 |

[a] Cells (2 × 10$^4$) were exposed to various doxorubicin concentrations for 96 h with the medium being replenished at 48 h. After 96 h the cells were detached by trypsinisation and counted using a Coulter counter. Inhibition of growth was calculated as a percentage of drug free control.

**Figure 4** Amplification of the *MDR1* (■) and *MDR2* (▩) genes in MCF-7/Dox, LP-gp and HP-gp cells over MCF-7/WT cells. Using the pHDR5A probe, which detects the *MDR1* and *MDR2* genes, or the *MDR2pvu*II probe, which detects only *MDR2*, copy numbers of the *MDR1* and *2* genes in each cell type were determined as described under Materials and methods. The results were quantitated using volume analysis on a phosphorimager. Equal DNA loading was assessed by hybridisation to a probe for the GAPDH gene which spanned bases 2 to 1023 of the GAPDH cDNA (Fort *et al.*, 1985).

**Figure 5** mRNA levels of *MDR1* (**a**) and *MDR2* (**b**) in MCF-7/WT (□), LP-gp (▨), MCF-7/Dox (■), and HP-gp (▩) cells. Levels of mRNA for *MDR1* and *MDR2* were determined using internally controlled RT–PCR, as described in Materials and methods. Results are the means ± s.d. of three determinations, except for *MDR1* in MCF-7/WT cells, which is the mean of two determinations.

MCF-7/WT cells. In LP-gp cells *MDR1* mRNA levels were $2.17 \pm 0.17$ amol $ng^{-1}$ RNA, which constitutes a 2500-fold increase over the MCF-7/WT cells. HP-gp cells contained $6.65 \pm 2.29$ amol $ng^{-1}$ RNA of *MDR1* mRNA, a 7556-fold increase. In HP-gp cells *MDR2* mRNA levels were $0.42 \pm 0.08$ amol $ng^{-1}$ RNA, 18 times the level observed in MCF-7/WT and LP-gp cells. MCF-7/Dox cells expressed levels of *MDR1* and *MDR2* mRNA that were intermediate between the levels of LP-gp and HP-gp cells, consistent with the ratio of LP-gp and HP-gp subpopulations in the parent MCF-7/Dox cell line.

*Analysis of PKC isozymes*

As PKC-α has been shown to play a role in the regulation of P-gp activity (Yu *et al.*, 1991; Ahmad and Glazer, 1993; Ahmad *et al.*, 1994) levels and subcellular distribution of PKC isozymes were examined. All three MCF-7 doxorubicin-resistant cell lines expressed PKC-α, -ζ, -ε and -θ (Figure 6 and Table II). Other PKC isozymes could not be detected by Western blot analysis. Total PKC expression was similar between LP-gp and HP-gp cells, but the isozyme distribution pattern in these cells differed substantially from that seen in

**Figure 6** Western blot analysis (**a**) and distribution (**b**) of PKC isozymes in MCF-7/WT, LP-gp and HP-gp cells. Distribution is expressed as a percentage of total cellular expression for each individual cell type. Expression was determined by Western blot analysis with the appropriate antibodies in the cytosolic (■), membrane (▧) and nuclear fractions (▩). Data for the MCF-7/Dox cells were very similar to those in LP-gp/HP-gp cells, and are not shown. Values are the means ± s.d. of three experiments, statistical significance was determined (*$P \leqslant 0.05$, **$P \leqslant 0.001$) by analysis of variance.

**Table II** PKC expression in MCF-7/WT cells and their drug-resistant counterparts

| *PKC* | *MCF-7/WT*[a] | *MCF-7/Dox* | *LP-gp* | *HP-gp* |
|---|---|---|---|---|
| α | +[b] | ++++ | ++++ | ++++ |
| θ | – | +++ | +++ | +++ |
| ε | ++++ | + | + | + |
| ζ | ++++ | ++ | ++ | ++ |

[a] Cells were harvested at near confluence and subcellular fractions were isolated. Analysis of protein levels in the cytosolic, membrane and nuclear fractions was by Western blotting with specific antibodies as described in Materials and methods. Antibody binding was detected using enhanced chemiluminescence and quantitated using a Molecular Dynamics densitometer. [b]Arbitrary measure based on consistent observations from three separate experiments. A representative blot is shown in Figure 6.

MCF-7/WT cells (Figure 6). Expression of PKC-α was undetectable in MCF-7/WT cells but it was found at identical levels in LP-gp and HP-gp cells. Most of the enzyme was localised in the cytosol, whereas 1% was associated with each of the membrane and nuclear compartments. Overall expression of PKC-ε and PKC-ζ in both LP-gp and HP-gp cells was decreased compared with MCF-7/WT cells (Table II). This decrease was mainly accounted for by a loss of cytosolic enzyme. However, in LP-gp and HP-gp cells PKC-ε was located to a greater extent in the nuclear and membrane fractions compared with MCF-7/WT cells. PKC-θ was not detectable in MCF-7/WT cells, but abundant in the resistant cells. In this case isozyme distribution was relatively homogeneous between the subcellular fractions.

### Discussion

The multidrug-resistant MCF-7/Dox cell line was originally derived in 1986 and has been widely used for studies of P-gp (Batist *et al.*, 1986). The results presented above show that our MCF-7/Dox cells comprised two subclones that were separable according to their ability to efflux R123. The presence of two clones in the MCF-7/Dox cells has been shown previously by MRK-16 antibody binding, but was not discussed (Yu *et al.*, 1991). In contrast another very recent analysis with MRK16 found only one cell clone (Molinari *et al.*, 1994).

In the work outlined above we present six separate pieces of evidence that suggest differences in P-gp expression levels between LP-gp and HP-gp cells that constitute the MCF-7/Dox cell line: (i) they accumulate and retain R123 differently; (ii) they demonstrate different levels of immunoreactivity with the anti-P-gp1 antibody MRK-16; (iii) they exhibit different degrees of *MDR1* and *MDR2* gene amplification; (iv) they display different sensitivity towards doxorubicin; (v) they possess different levels of *MDR1* and *MDR2* mRNA; and (vi) they are differentially susceptible towards verapamil.

*MDR1* mRNA levels were 2500- and 7556-fold greater in the LP-gp and HP-gp cells respectively, than in MCF-7/WT cells. The difference in *MDR1* mRNA levels between LP-gp and HP-gp cells was therefore only 3-fold. However, both R123 accumulation and MRK16 binding indicate a difference of approximately one order of magnitude in the amount of P-gp1 protein between LP-gp and HP-gp cells. Therefore a post-translational regulatory step for P-gp1 stability may be operative in HP-gp cells. Both *MDR1* and *2* genes were amplified in LP-gp cells about 9- and 8-fold respectively, over MCF-7/WT cells. In LP-gp cells this amplification translated into an increase of *MDR1* mRNA level of 2500, whereas *MDR2* mRNA was not increased. This discrepancy indicates that a regulatory step was involved in the control of the *MDR1* gene in which the rate of gene transcription or mRNA half-life was increased about 250-fold over MCF-7/WT cells to result in the observed mRNA level. The result with *MDR2* is more difficult to analyse, given the possibility that the gene may not have been amplified intact. In the case of HP-gp cells the picture was similar. *MDR1* and *MDR2* gene amplification in HP-gp cells was 78- and 65-fold respectively, over MCF-7/WT cells. However, *MDR1* and *MDR2* mRNA levels were increased 7556- and 18-fold respectively. In analogy to LP-gp cells this difference indicates an increase in *MDR1* gene transcription rate or mRNA half-life of about 100-fold over MCF-7/WT cells. An increase in *MDR1* transcription rate has been previously observed in MCF-7/Dox, doxorubicin-resistant ovarian 2780 and human colon carcinoma SW620 cells (Morrow *et al.*, 1992; Madden *et al.*, 1993). These data suggest that regulation of mRNA level in the LP-gp and HP-gp cells may occur to a greater degree at the transcriptional rather than post-transcriptional level.

Correlation of the results obtained by fluorescence analysis using the MRK-16 antibody with those observed for R123 accumulation suggests that R123 efflux was mediated completely via P-gp. Furthermore, as MRK-16 is specific for the P-gp1 isoform (Schinkel *et al.*, 1991, 1993), we conclude that R123 efflux is driven by P-gp1 and not P-gp2, the mRNA for which was increased in HP-gp but not LP-gp cells. This result is consistent with that previously obtained by Ludescher *et al.* (1993) in B-cell chronic lymphocytic leukaemia. All MCF-7-derived cell clones had detectable *MDR2* mRNA levels. The correlation between R123 accumulation and P-gp1 levels as detected by MRK 16 in LP-gp and HP-gp cells indicates that there is no additional post-translational regulation of P-gp protein activity in HP-gp in comparison with LP-gp cells.

As cPKC-α has been shown to play an important role in the post-translational regulation of P-gp activity (Yu *et al.*, 1991; Ahmad and Glazer, 1993; Ahmad *et al.*, 1994), we analysed the level and distribution of PKC isozymes. In LP-gp and HP-gp cells overall levels of PKC isozymes -α, -ε, -ζ and -θ, were similar. However, compared with MCF-7/WT cells the resistant cells expressed higher levels of cPKC-α and nPKC-θ and lower levels of nPKC-ε and aPKC-ζ. The subcellular distribution of nPKC-ε differed significantly between LP-gp, HP-gp and MCF-7/WT cells. The resistant cells possessed more nPKC-ε in the membrane, and especially the nucleus, than wild-type cells. The fact that this distribution pattern locates nPKC-ε more efficiently in close vicinity to P-gp is commensurate with a role for this enzyme in the post-translational regulation of P-gp1 activity. This finding is consistent with the report of Slapak *et al.*, 1993, who observed a decrease in overall PKC activity in the cytosol of HL-60 cells with acquired vinblastine resistance and an increase in enzyme content in the membrane fractions compared with HL-60 WT cells. The results presented above show for the first time that PKC-θ is present in MCF-7/Dox cells and its subclones. Its absence in MCF-7/WT cells and expression in all three cell compartments, particularly in the membrane and nuclear fractions of LP-gp and HP-gp cells, render it another candidate for post-translational regulation of P-gp activity.

In conclusion we derived two cell lines from the widely used MCF-7/Dox cell line, of the same origin, which differed in their expression of both *MDR1* and *MDR2* genes. This difference translated into an altered level of P-gp1 expressed in the plasma membrane and a corresponding difference in the ability of each cell line to efflux R123. In both LP-gp and HP-gp cells the change in *MDR2* mRNA over MCF-7/WT cells was smaller than the increase in *MDR2* gene copy number indicating a decreased transcription rate or mRNA stability or defective amplicon. The opposite was the case for the *MDR1* gene in which the increase in *MDR1* mRNA level was greater than that which could be accounted for by increased gene copy number. This result indicates an increased rate of *MDR1* gene transcription or mRNA stability. Comparison of MRK-16 binding with R123 retention in each cell type is consistent with the hypothesis that R123 efflux occurs only through P-gp1. Comparison of P-gp protein level and mRNA levels between LP-gp and HP-gp cells suggested that in addition to the transcriptional/post transcriptional regulation of *MDR1* expression some post-translational increase in protein stability occurred.

However, the identical 10-fold difference in R123 accumulation and P-gp levels between LP-gp and HP-gp cells indicated no change in post-translational regulation of protein activity. Analysis of PKC isozymes indicated a dramatic increase in nPKC-$\alpha$ and nPKC-$\theta$ levels in both LP-gp and HP-gp cells over MCF-7/WT cells. In comparison with MCF-7/WT cells nPKC-$\varepsilon$ expression was reduced overall in the resistant cell types, however the enzyme was distributed differently. More nPKC-$\varepsilon$ was located in the membrane and nucleus, which might indicate an important role for nPKC-$\varepsilon$ in the regulation of P-gp1 activity.

Overall, the results highlight the complicated and multifaceted nature of regulation of the multidrug resistance phenotype in MCF-7/Dox cells. A better appreciation of how it is controlled will help to devise more efficacious therapies to overcome P-gp-mediated drug resistance.

### References

AHMAD S AND GLAZER RI. (1993). Expression of the antisense cDNA for protein kinase C alpha attenuates resistance in doxorubicin-resistant MCF-7 breast carcinoma cells. *Mol. Pharmacol.*, **43**, 858-862.

AHMAD S, SAFA AR AND GLAZER RI. (1994). Modulation of P-glycoprotein by protein kinase C alpha in a baculovirus expression system. *Biochemistry*, **33**, 10313-10318.

BATES SE, LEE JS, DICKSTEIN B, SPOLYAR M AND FOJO AT. (1993). Differential modulation of P-glycoprotein transport by protein kinase C inhibition. *Biochemistry*, **32**, 9156-9164.

BATIST G, TULPULE A, SINHA BK, KATKI AG, MYERS C AND COWAN KH. (1986). Overexpression of a novel anionic glutathione transferase in multidrug-resistant human breast cancer cells. *J. Biol. Chem.*, **261**, 15544-15549.

BECK W, MUELLER TJ AND TANZER LR. (1979). Altered surface membrane glycoproteins in vinca alkaloid-resistant human leukaemic lymphoblasts. *Cancer Res.*, **39**, 2070-2076.

BRADFORD MM. (1976). A rapid and sensitive method for the quantitation of microgram quantities of protein utilizing the principle of protein dye binding. *Anal. Biochem.*, **72**, 248-254.

BROWN PC, THORGEIRSSON SS AND SILVERMAN JA. (1993). Cloning and regulation of the rat *mdr2* gene. *Nucleic. Acids Res.*, **21**, 3885-3891.

CHAMBERS TC, MCAVOY EM, JACOBS JW AND EILON G. (1990). Protein kinase C phosphorylates P-glycoprotein in multidrug resistant human KB carcinoma cells. *J. Biol. Chem.*, **265**, 7679-7686.

CHAMBERS TC, ZHENG B AND KUO JF. (1992). Regulation by phorbol ester and protein kinase C inhibitors and by a protein phosphatase inhibitor (okadaic acid), of P-glycoprotein phosphorylation and relationship to drug accumulation in multidrug-resistant human KB cells. *Mol. Pharmacol.*, **41**, 1008-1015.

CHAN HSL, BRADLEY G, THORNER P, HADDAD G, GALLIE BL AND LING V. (1988). A sensitive method for immunocytochemical detection of P-glycoprotein in multidrug resistant human ovarian carcinoma cell lines. *Methods Lab. Invest.*, **59**, 870-875.

CHAUDHARY PM AND RONINSON IB. (1992). Activation of MDR1 (P-glycoprotein) gene expression in human cells by protein kinase C agonists. *Oncology Research*, **4**, 281-290.

CHIN JE, SOFFIR R, NOONAN KE, CHOI K AND RONINSON IB. (1989). Structure and expression of the human *MDR* (P-glycoprotein) gene family. *Mol. Cell. Biol.*, **9**, 3808-3820.

CHOI K, FROMMEL THO, STERN RK, PEREZ CF, KRIEGLER M, TSURUO T AND RONINSON IB. (1991). Multidrug resistance after retroviral transfer of the human MDR1 gene correlates with P-glycoprotein density in the plasma membrane and is not affected by cytotoxic selection. *Proc. Natl Acad. Sci. USA*, **88**, 7386-7390.

COWAN KH, BATIST G, TUPULE A, SINHA BK AND MYERS CE. (1986). Similar biochemical changes associated with multidrug resistance in human breast cancer cells and carcinogen-induced resistance to xenobiotics in rats. *Proc. Natl Acad. Sci. USA*, **83**, 9328-9332.

CURRIER SJ, KANE SE, WILLINGHAM MC, CARDARELLI CO, PASTAN I AND GOTTESMAN MM. (1992). Identification of residues in the first cytoplasmic loop of P-glycoprotein involved in the function of chimeric human *MDR1-MDR2* transporters. *J. Biol. Chem.*, **267**, 25153-25159.

EFFERTH T AND OSIEKA R. (1993). Clinical relevance of the MDR-1 gene and its gene product, P-glycoprotein, for cancer chemotherapy: A meta analysis. *Tumor Diagnostik und Therapie*, **14**, 238-243.

ENDICOTT JA AND LING V. (1989). The biochemistry of P-glycoprotein mediated multidrug resistance. *Ann. Rev. Biochem.*, **58**, 137-171.

FAIRCHILD CR, KAO-SHAN CHIEN-S, WANG-PENG J, ROSEN N, ISRAEL MA, MALERA PW, COWAN KH AND GOLDSMITH ME. (1987). Isolation of amplified and overexpressed DNA sequences from adriamycin resistant human breast cancer cells. *Cancer Res.*, **47**, 5141-5148.

FINE RL, PATEL J AND CHABNER BA. (1988). Phorbol esters induce multidrug resistance in human breast cancer cells. *Proc. Natl Acad. Sci. USA*, **85**, 582-586.

FORT P, MARTY L, PIECHAEZYL M, SABROUTY SE, DANI C, JEANTEUR P AND BLANCHARD JM. (1985). Various rat tissues express only one major mRNA species from the glyceraldehyde-3-phosphate-dehydrogenase multigenic family. *Nucleic Acids Res.*, **13**, 1431-1443.

FUTSCHER BW, BLAKE LL, GERLACH JH, GROGAN TIM AND DALTON WS. (1993). Quantitative polymerase chain reaction analysis of mdr1 mRNA in multiple myeloma cell lines and clinical specimens. *Anal. Biochem.*, **213**, 414-421.

GANT TW, SILVERMAN JA, BISGAARD HC, BURT RK, MARINO PA AND THORGEIRSSON SS. (1991). Regulation of 2-acetylamino-fluorene-and 3-methylcholanthrene-mediated induction of multidrug resistance and cytochrome P450IA gene family expression in primary hepatocyte cultures and rat liver. *Mol. Carcinog.*, **4**, 499-509.

GANT TW, SILVERMAN JA AND THORGEIRSSON SS. (1992). Regulation of P-glycoprotein expression in hepatocyte cultures and liver cell lines by a *trans*-acting transcriptional repressor. *Nucleic Acids Res.*, **20**, 2841-2846.

GREIF H, BEN-CHAIM J, SHIMON T, BECHOR E, ELDAR H AND LIVNEH E. (1992). The protein kinase C related PKC-L (Eta) gene product is localized to cell nucleus. *Mol. Cell. Biol.*, **12**, 1304-1311.

KARTNER N, RIORDAN JR AND LING V. (1983*a*). Cell surface P-glycoprotein associated with multidrug resistance in mammalian cell lines. *Science*, **221**, 1285-1288.

KARTNER N, SHALES M, RIORDAN JR AND LING V. (1983*b*). Daunorubicin-resistant chinese hamster ovary cells expressing multidrug resistance and cell surface P-glycoprotein. *Cancer Res.*, **43**, 44143-4419.

KOHNO K, TANIMURA H, SATO S, NAKAYAMA Y, MAKINO Y, WADA M, FOJO AT AND KUWANO M. (1994). Cellular control of human multidrug resistance 1 (*mdr-1*) gene expression in absence and presence of gene amplification in human cancer cells. *J. Biol. Chem.*, **269**, 20503-20508.

LEE CH, BRADLEY G, ZHANG JT AND LING V. (1993). Differential expression of P-glycoprotein genes in primary rat hepatocyte culture. *J. Cell. Physiol.*, **157**, 392-402.

LUDESCHER C, HILBE W, EISTERER W, PREUSS E, HUBER C, GOTWALD M, HOFMANN J AND THALER J. (1993). Activity of P-glycoprotein in B-cell chronic lymphocytic leukemia determined by a flow cytometric assay. *J. Natl Cancer Inst.*, **85**, 1751-1758.

MA L, MARQUARDT D, TAKEMOTO L AND CENTER MS. (1991). Analysis of P-glycoprotein phosphorylation in HL60 cells isolated for resistance to vincristine. *J. Biol. Chem.*, **266**, 5593-5599.

MADDEN MJ, MORROW CS, NAKAGAWA M, GOLDSMITH ME, FAIRCHILD CR AND COWAN KH. (1993). Identification of 5′ and 3′ sequences involved in the regulation of transcription of the human mdr1 gene in vivo. *J. Biol. Chem.*, **268**, 8290-8297.

MOLINARI A, CIANFRIGLIA M, MESCHINI S, CALCABRINI A AND ARANCIA G. (1994). P-glycoprotein expression in the Golgi apparatus of multidrug-resistant cells. *Int. J. Cancer*, **59**, 789-795.

MORROW CS, CHIU J AND COWAN KH. (1992). Posttranscriptional control of glutathione S-transferase $\pi$ gene expression in human breast cancer cells. *J. Biol. Chem.*, **267**, 10544–10550.

NG WF, SARANGI F, ZASTAWNY RL, VEINOT-DREBOT L AND LING V. (1989). Identification of members of the P-glycoprotein multigene family. *Mol. Cell. Biol.*, **9**, 1224–1232.

RIORDAN JR AND LING V. (1979). Purification of P-Glycoprotein from plasma membrane vesicles of chinese hamster ovary cell mutants with reduced colchicine permeability. *J. Biol. Chem.*, **254**, 12701–12705.

SAMBROOK J, FRITSCH EF AND MANIATIS T. (1989). *Molecular Cloning*, 2nd edn, Vol. 1–3. Cold Spring Harbor Laboratory Press: Cold Spring Harbor, NY.

SCHINKEL AH, ROELOFS MEM AND BORST P. (1991). Characterization of the human *MDR3* P-glycoprotein and its recognition by P-glycoprotein-specific antibodies. *Cancer Res.*, **51**, 2628–2635.

SCHINKEL AH, ARCECI RJ, SMIT JJM, WAGENAAR E, BAAS F, DOLLE M, TSURUO T, MECHETNER EB, RONINSON IB AND BORST P. (1993). Binding properties of monoclonal antibodies recognizing external epitopes of the human MDR1 P-glycoprotein. *Int. J. Cancer*, **55**, 478–484.

SHEN D, CARDARELLI C, HWANG J, CORNWELL MM, RICHERT N, ISHI S, PASTAN I AND GOTTESMAN MM. (1986*a*). Multiple drug resistant human KB carcinoma cells independently selected for high-level resistance to colchicine, adriamycin or vinblastine show changes in expression of specific proteins. *J. Biol. Chem.*, **261**, 7762–7770.

SHEN D-W, FOJO A, CHIN JE, RONINSON IB, RICHERT N, PASTAN I AND GOTTESMAN MM. (1986*b*). Human multidrug-resistant cell lines: Increased *mdr*1 expression can precede gene amplification. *Science*, **232**, 643–645.

SLAPAK CA, KHARBANDA S, SALEEM A AND KUFE DW. (1993). Defective translocation of protein kinase C in multidrug-resistant HL-60 cells confers a reversible loss of phorbol ester-induced monocytic differentiation. *J. Biol. Chem.*, **268**, 12267–12273.

STANWELL C, GESCHER A, BRADSHAW TD AND PETTIT GR. (1994). The role of protein kinase C isoenzymes in the growth inhibition caused by bryostatin 1 in human A549 lung and MCF-7 breast carcinoma cells. *Int. J. Cancer*, **56**, 585–592.

UEDA K, CARDARELLI C, GOTTESMAN MM AND PASTAN I. (1987). Expression of a full length cDNA for the human 'MDR1' gene confers resistance to colchicine, doxorubicin and vinblastine. *Proc. Natl Acad. Sci. USA*, **84**, 3004–3008.

VAN DER BLIEK AM AND BORST P. (1989). Multidrug Resistance. *Adv. Cancer Res.*, **52**, 165–203.

YU G, AHMAD S, AQUINO A, FAIRCHILD CR, TREPEL JB, OHNO S, SUZUKI K, TSURO T, COWAN KH AND GLAZER RI. (1991). Transfection with protein kinase C$\alpha$ confers increased multidrug resistance to MCF-7 cells expressing P-glycoprotein. *Cancer Communications*, **3**, 181–189.

ZHANG F, RILEY J AND GANT TN. (1996). Use of internally controlled reverse transcriptase–polymerase chain reaction for absolute quantitation of individual multidrug resistance gene transcripts in tissue samples. *Electrophoresis*, **17**, (in press).